# A Statistical Model of Protein Sequence Similarity and Function Similarity Reveals Overly-Specific Function Predictions

**DOI:** 10.1371/journal.pone.0007546

**Published:** 2009-10-21

**Authors:** Brenton Louie, Roger Higdon, Eugene Kolker

**Affiliations:** 1 Bioinformatics and High-throughput Analysis Laboratory, Seattle Children's Research Institute, Seattle, Washington, United States of America; 2 Predictive Analytics, Seattle Children's Hospital, University of Washington School of Medicine, Seattle, Washington, United States of America; 3 Biomedical and Health Informatics Division, Department of Medical Education and Biomedical Informatics, University of Washington School of Medicine, Seattle, Washington, United States of America; NERC Centre for Ecology and Hydrology, United Kingdom

## Abstract

**Background:**

Predicting protein function from primary sequence is an important open problem in modern biology. Not only are there many thousands of proteins of unknown function, current approaches for predicting function must be improved upon. One problem in particular is overly-specific function predictions which we address here with a new statistical model of the relationship between protein sequence similarity and protein function similarity.

**Methodology:**

Our statistical model is based on sets of proteins with experimentally validated functions and numeric measures of function specificity and function similarity derived from the Gene Ontology. The model predicts the similarity of function between two proteins given their amino acid sequence similarity measured by statistics from the BLAST sequence alignment algorithm. A novel aspect of our model is that it predicts the degree of function similarity shared between two proteins over a continuous range of sequence similarity, facilitating prediction of function with an appropriate level of specificity.

**Significance:**

Our model shows nearly exact function similarity for proteins with high sequence similarity (bit score >244.7, e-value >1e^−62^, non-redundant NCBI protein database (NRDB)) and only small likelihood of specific function match for proteins with low sequence similarity (bit score <54.6, e-value <1e^−05^, NRDB). For sequence similarity ranges in between our annotation model shows an increasing relationship between function similarity and sequence similarity, but with considerable variability. We applied the model to a large set of proteins of unknown function, and predicted functions for thousands of these proteins ranging from general to very specific. We also applied the model to a data set of proteins with previously assigned, specific functions that were electronically based. We show that, on average, these prior function predictions are more specific (quite possibly overly-specific) compared to predictions from our model that is based on proteins with experimentally determined function.

## Introduction

Protein functional prediction, or *annotation*, remains an important open problem in biology [1]–[23]. Of the millions of proteins residing in public repositories only a small percentage have had their functions determined experimentally [2], [24]. The vast majority of proteins have been annotated through predictive methods which work by comparing protein sequences and determining their degree of similarity. This is carried out by computer programs such as BLAST [25], [26] or various other tools and databases [27]–[33]. This process, where a protein of unknown function receives the function from a known protein, has been described as “annotation transfer” [7], [8], [18]. The rationale being that proteins of similar sequence fold into similar protein structures which therefore perform similar biological functions. However, in spite of much research more needs to be done to improve the accuracy of function prediction [1]–[3], [5], [34], [35], [9], [10], [12], [36]–[38], [22], [39]. There is also a huge and burgeoning population of “hypothetical” proteins with only moderate similarity to proteins of known function. Limitations of current approaches in this moderate similarity range, or “twilight zone”, make it extremely difficult to annotate proteins of this type reliably [40], [5], [41], [14], [17]. An important part of the annotation puzzle that is missing in particular is an in-depth understanding of the relationship between sequence similarity and function similarity over a continuous range and the amount of variability inherent in the relationship over all ranges of sequence similarity. Solving this puzzle requires generation of a sufficiently large and diverse data set of proteins with experimentally characterized function, determining the best way to represent function for modeling purposes, and both appropriately building and applying a proper statistical model.

To address this challenge we present here a novel annotation model to predict the function of a protein of unknown function based on its sequence similarity to a protein of known function. Our annotation model is trained on proteins whose functions have been experimentally characterized and is therefore based on primary biological evidence. A major concern with most existing protein annotations is that they are predicted computationally and not derived experimentally [2], [10]. Previous approaches for predicting function which use these data can lead to “circular logic”, i.e. using predictions for prediction. Consequences of this can be over-prediction (otherwise called overly-specific prediction) [1], or outright erroneous predictions. It is therefore imperative that any statistical model be based on primary biological evidence.

In our annotation model, BLAST sequence similarity statistics serve as the *predictor* variables. The output of the model, or the *response* variable, is a measure of function similarity and represents a novel aspect of our approach. The output provides a real numbered value of the similarity of the functional match between two proteins as opposed to just a textual protein function description provided in a typical annotation by BLAST. This numerical measure is enabled by the Gene Ontology (GO) [42]. The GO is a rich, hierarchical description of molecular protein functions structured as a directed acyclic graph. Child, or descendant, terms of the top level or “root” node (called “molecular function”) become increasingly specific in their description of protein function. This structure allows for the measurement of distance between GO terms and development of a numerical measure to represent both function specificity and function similarity (see Methods).

We evaluated a couple of measures of function specificity which are possible in the GO. The first is the level, or *depth*, of a GO term. There are idiosyncrasies in the GO however which make GO term depth problematic. Path lengths from the root to any particular GO term are highly variable (2–14 levels), which makes it difficult to compare specificity between terms using this metric [43]. A better, more normalized measure is *Information Content* (IC) [43]. IC is related to the probability of occurrence of a particular GO term in a data set where less common terms have higher IC, which is interpreted as being more specific. In general, the IC of GO terms monotonically increase as the GO hierarchy is traversed upward and the root term always carries an IC of 0.0. Based on IC, metrics can be developed to measure the level of function similarity between two proteins. Having this numerical measure of function specificity enables our statistical model to make predictions about function specificity and function similarity between GO terms (see Methods and Supporting Figure S1).

The model described here serves as a novel tool for protein annotation by predicting the specificity of function, based on the GO hierarchy, which may be shared between two proteins for a given level of sequence similarity. Through the statistical modeling process we shed light on the variability in the relationship between sequence similarity and function similarity. In addition, we demonstrate the usefulness of our model through two use cases: evaluating existing protein functional annotations based on predictive methods currently residing in protein databases and providing possible annotations for thousands of hypothetical proteins.

## Results

### Building gold-standard data sets

We created a “gold-standard” training and test data sets as a first step in developing the annotation model. The training and test set were created using only single function proteins from RefSeq and Uniprot which were experimentally characterized (those containing “IDA” GO evidence codes, see Methods). This resulted in 425 proteins from RefSeq for the training set, and 313 proteins from Uniprot for the test set which was used to validate models. All proteins within each set were aligned against each other using BLAST, this resulted in 2091 alignments being returned for the training set and 2055 alignments for the test set (see Methods).

### Numerical measures of function specificity

**Figure 1:**
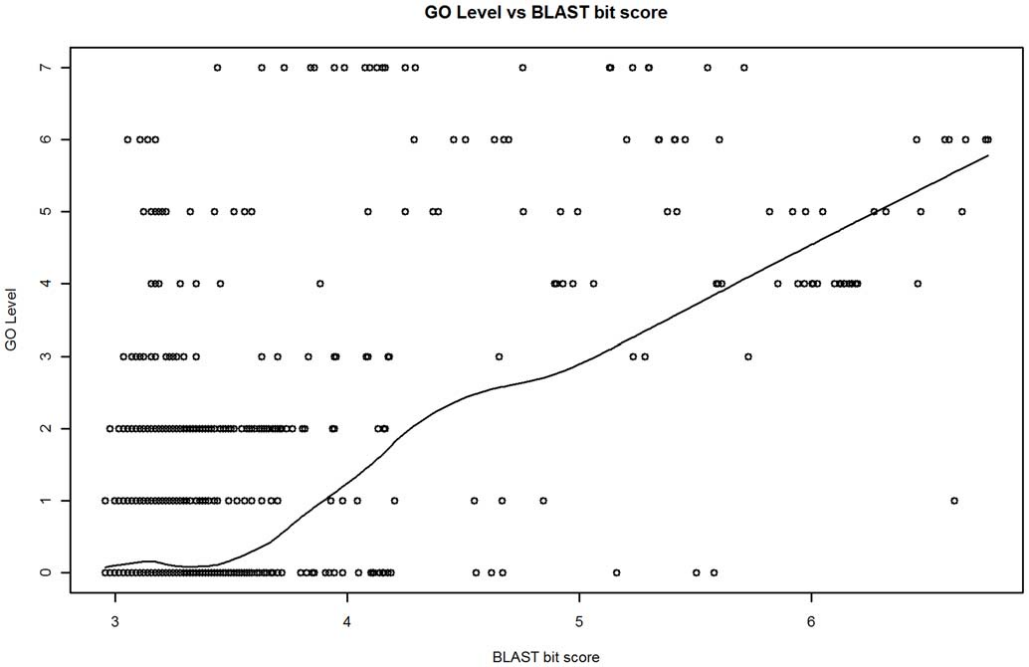
BLAST bit score (log) vs GO term depth. The trend shown is a lowess line. GO level generally increases with higher bit scores, however there is a high degree of variability in GO level over all ranges of bit scores, even for bit scores above 6.0 which indicate a high degree of sequence similarity.

**Figure 2:**
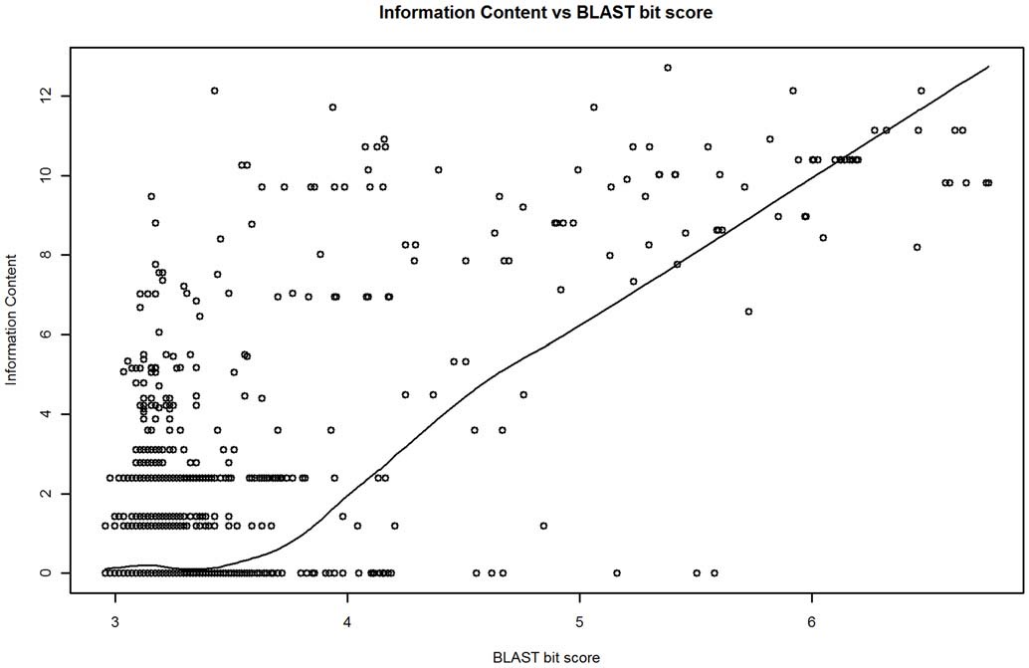
BLAST bit score (log) versus IC. The trend shown is a lowess line. The IC of GO terms generally increases with higher bit scores. IC is less variable than GO level across most bit score ranges, however there remains a significant degree of variability even above a bit score of 6.0.

**Figure 3:**
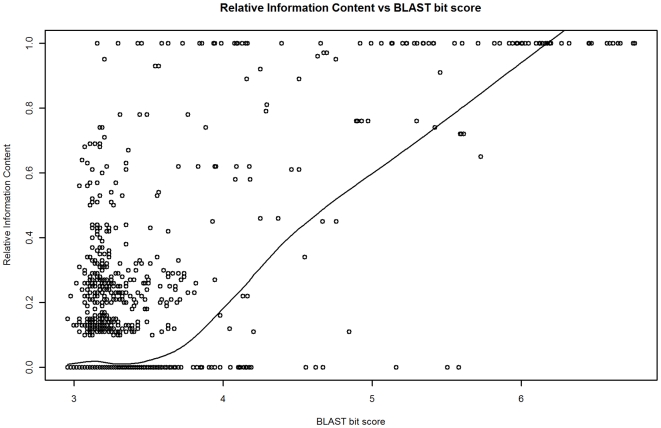
BLAST bit score (log) versus RIC. The trend shown is a lowess line. The RIC statistic normalizes the variability of IC values in the training data. RIC is the least variable statistic across most bit score ranges. This is especially so for bit score ranges above 6.0, where all RIC values are 1.0 (no variability).

GO term depth and IC are measures of function specificity for a single GO term. However, the purpose of our annotation model is to output a measure of the relationship between two GO terms (assigned to the two proteins in a BLAST comparison). We call the relationship between two GO terms the *function similarity*. Three measures of this were considered: 1) GO term depth of the common ancestral GO term for the GO terms assigned to the two proteins in a BLAST alignment, 2) the IC of the common ancestral GO term, and 3) the *Relative Information Content* (RIC). RIC is the ratio of the IC of the common ancestral GO term and the mean IC of the GO terms for two proteins in a BLAST alignment (see Methods). Whereas IC has less variability and a stronger relationship with BLAST bit score than GO term depth (adjusted R^2^ of 0.47 versus 0.34 respectively), normalizing IC by generating the RIC metric reduces the influence on the model of the variability of IC values in the training data (which improves prediction accuracy). The reduction in variability is especially apparent for log bit scores greater than 6.0 (training data). In this bit score range GO level has a coefficient of variation (CV) of 23.6 (Figure 1), whereas IC is less variable (CV = 9.6, Figure 2). The effectiveness of normalizing IC by using RIC however can be clearly seen as RIC has CV of 0.0 in this range (Figure 3). For bit score ranges below 4.0, the CV's are comparable (186.4, 188.5, and 188.9) for GO level, IC, and RIC respectively.

Knowledge of the IC of the GO term assigned to proteins is still useful in regards to statistical modeling in that proteins determined to be annotated with non-specific functions can be removed from the data set. Non-specific functions, such as the GO terms *“protein binding”* or *“catalytic activity”*, confounds the process of statistical modeling due to the high numbers of proteins annotated with these terms (2566 and 5021 proteins respectively) which bias the data sets. In addition, non-specific terms do not reflect the true function of proteins. Consider that the true function of a protein with a non-specific GO term can be a single specific GO term among many possible child terms.

### Statistical Model Selection

**Figure 4:**
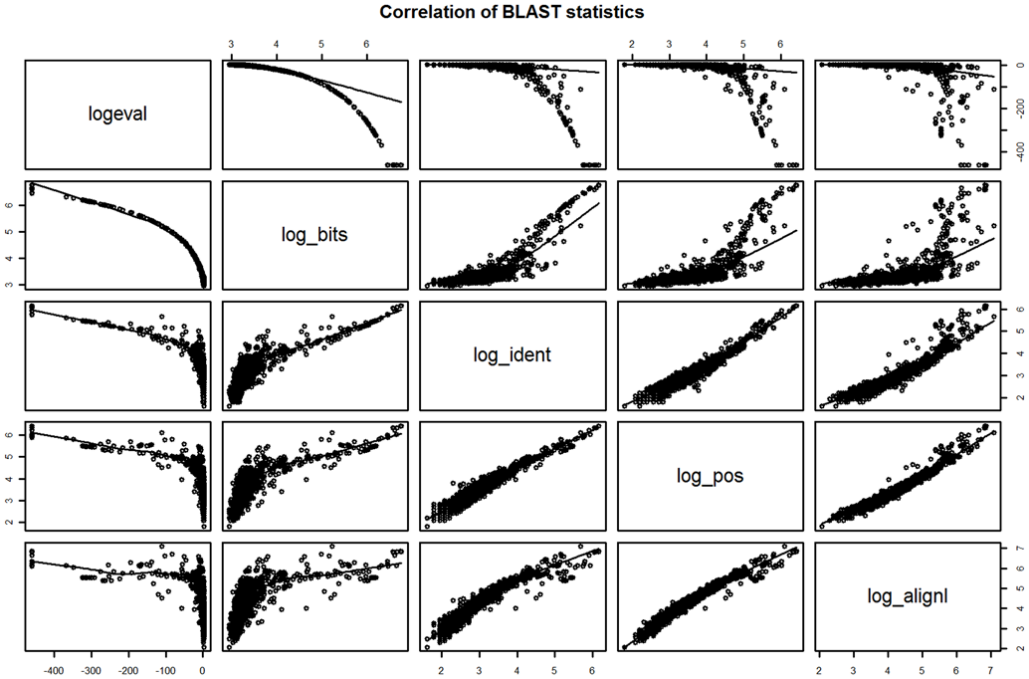
Correlation between log-transformed BLAST statistics. BLAST statistics are generally highly correlated with each other. If two variables are highly correlated the information they provide about a response variable (i.e. RIC) is not independent. Generally only one of the variables in this case will add significant predictive power to a statistical model.

Generalized Linear and Generalized Additive models (GLM and GAM respectively) were created using all log-transformed BLAST statistics as predictor variables (“full” models) and also using “stepwise” functions (“step” models) which iteratively build models based on the Akaike Information Criterion (AIC, see Methods). Stepwise models retained only two predictor variables: 1) bit score, and 2) max length. (The “max length” variable is the length of either the query or subject sequence in a BLAST alignment, whichever is longer). We found that bit score was the most influential and significant predictor of RIC for GLM and GAM models built stepwise or the full complement of BLAST statistics. The well-known e-value statistic was not used since it is a composite of the bit score and other statistics related to sequence lengths, and is meant more for the context of database search [44]. E-value is an estimate of the number of “hits” due to chance for a given database size rather than an error measure of individual pair-wise alignments as is of interest here. There is also a high correlation between BLAST statistics which indicates that they carry similar information and therefore add little subsequent predictive capability to a statistical model. This is illustrated in a pair-wise plot of selected BLAST statistics (Figure 4). Given that bit score was the most influential, single parameter GLM and GAM models (“single” models) were also created using only the bit score as predictor. Statistical tests comparing the full models with the step and single models indicated that differences in model fits to the training data were not significant. This is indicated by non-significant reductions in model deviance in all cases: 1) p = 0.12 and 0.93 between GLM models, and 2) p = 0.14 and 0.66 between GAM models.

**Figure 5:**
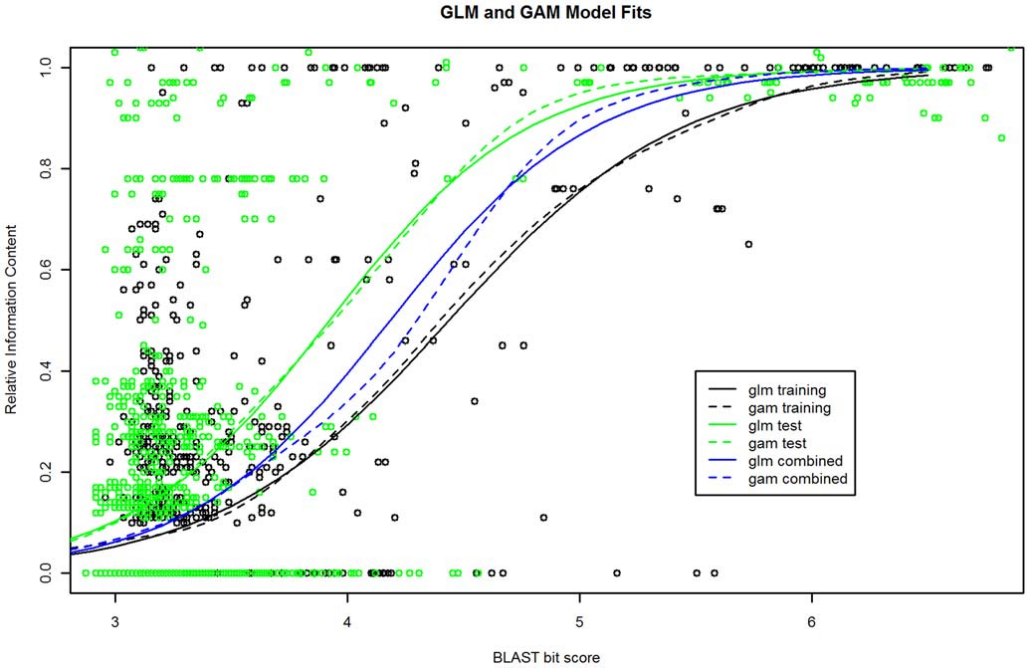
GLM and GAM model fits on the Training, Test, and Combined data sets. Functional similarity (RIC) between two proteins generally increases for higher similarity levels, measured by bit score. The RIC predictions for the GLM and GAM model fits to the Test data (solid and dashed green lines) are somewhat higher than the GLM and GAM model fits to the Training data (solid and dashed black lines), indicating some bias in the data sets. GLM and GAM models fits using a combined data set (training + test) may be more general for prediction (solid and dashed blue lines). There is not a significant difference between the GAM and GLM model fits on any data set.

Differences between single GLM and GAM models were also non-significant (p = 0.31), indicating little difference in predictive capability between the GLM model and the more complex GAM model. Plots of model fits for the single GLM and GAM models on training and test data sets to illustrate relationship between RIC and BLAST bit score can be seen in Figure 5. Fits for both types of models are very similar. Overall, RIC increases with increasing bit score but is extremely variable in bit score ranges below about 4.0 (bit score ∼54.6, e-value ∼1e^−05^, NRDB) indicating that predicting RIC with any precision is difficult in this range, for any statistical modeling approach. For bit score ranges above about 5.5–6.0 (bit score ∼244.7, e-value ∼1e^−62^, NRDB) RIC values are very close to 1.0 with little variation (Figure 3), indicating that it, and specific protein function, can be predicted with high precision.

### Statistical Model Prediction Error on Test Data

Model prediction error was calculated by Mean Squared Error (MSE) and Mean Residual Deviance (MRD) (see Methods). The MRD error statistic was included in the model evaluation process since it accounts for the observed unequal variance in RIC (Figure 5). MSE does not weight observations for unequal variance. Calculations of model prediction error on the training data indicated little or no improvement in model performance when more predictor variables are included, i.e. models with all predictor variables (full), or built stepwise (step). However, estimates of prediction error on the test data set produce different results from those on the training set (Table 1). As to be expected, the prediction error was considerably larger for the test set compared to the training set. The full models perform slightly worse than the stepwise or single models, an example of potential over-fitting (i.e. not generalizing well to a new data set). However, the difference in prediction error between the single and stepwise models for both the GLM and GAM method are in general very small. These results indicate that the single variable GLM model is the best choice based on its simplicity and performance.

**Table 1:** Prediction error of GLM (glm.*) and GAM (gam.*) models on training and test data sets.

Model	MSE (Train)	MRD (Train)	MSE (Test)	MRD (Test)
glm.full	0.032	0.277	0.082	0.511
glm.step	0.030	0.278	0.081	0.499
glm.bits	0.032	0.283	0.082	0.497
gam.full	0.030	0.264	0.085	0.534
gam.step	0.030	0.274	0.081	0.498
gam.bits	0.032	0.281	0.082	0.497

Results are shown for models with three sets of predictor variables: 1) full models which contain all BLAST statistics (full), 2) stepwise models which contain BLAST statistics selected during using a stepwise AIC variable selection process (step), and 3) bits models only utilize the BLAST bit score (log) as a predictor variable (bits). Models were assessed using both MSE and MRD (lower values are better). On the training set, models with all predictor variables (glm.full, and gam.full) fit the data best (MSE = 0.032 and 0.030 and MRD = 0.277 and 0.264 respectively). However, models with more predictor variables do not perform significantly better on the test data versus models which have bit score as a single predictor.

### Statistical Model Use Cases

The annotation model was used to predict function similarities between proteins of interest and the data sets of proteins with gold-standard annotations in our training and test sets. This scenario is analogous to both annotating proteins of unknown function (hypotheticals) and analyzing existing functional annotations created using electronic methods, for proteins currently residing in the RefSeq and Uniprot databases. These types of scenarios are relevant to current challenges in modern biology regarding improving the accuracy of function predictions and annotation of the large population of hypothetical proteins.

### Use Case 1: Electronic versus Experimental Annotations

**Figure 6:**
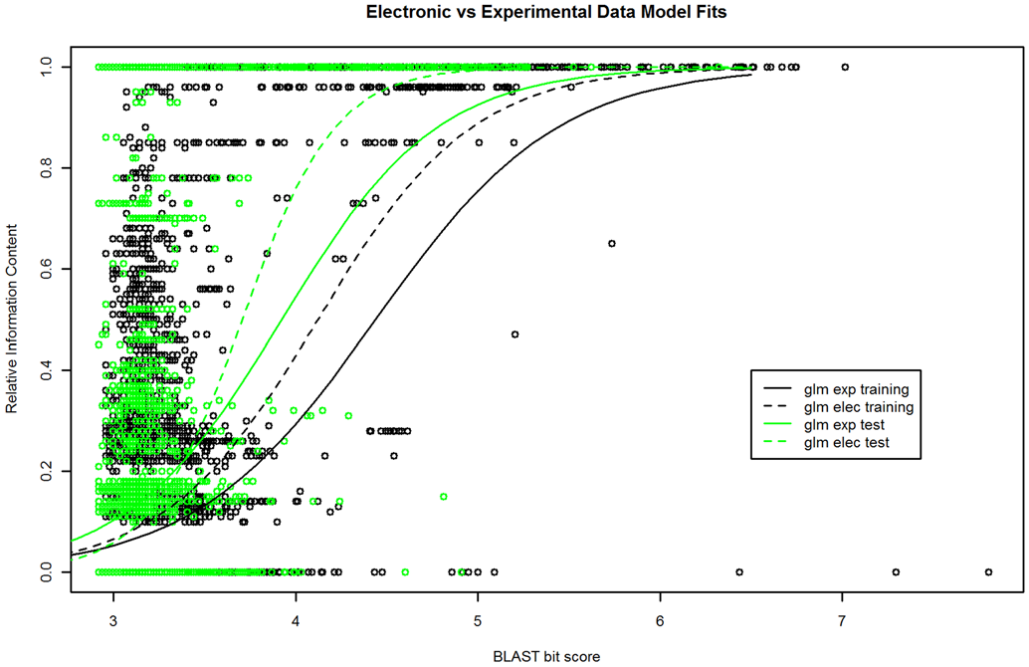
GLM model fits from BLAST alignments generated from proteins with experimental functions only and from proteins with electronic annotations. The GLM models fit on data containing only experimental annotations (solid lines) predict a lower RIC for most ranges of bit scores than for models fit using electronic annotations (dashed lines), for both the Training (black lines) and Test (green lines) data sets. The difference in predicted RIC is greatest for (log) bit score ranges of about 4.0 to 5.0 (bit scores 54.6 to 148.4, e-values 1e^−05^ to 1e^−34^, NRDB).

We compared RIC predictions from the model trained on proteins with experimental functions to RIC predictions based on electronic annotation data. BLAST alignments were obtained for a set of electronically annotated proteins from RefSeq and Uniprot versus the experimental data sets (see Methods). Electronically-based models predict the RIC from BLAST bit scores using the same type of single variable GLM model. RIC predictions from the model based on experimental functions were plotted against RIC predictions from the model based on electronic functions (Figure 6). RIC predictions from the experimental model are generally *lower* than those predicted by the electronic model. This is especially so for log bit scores 4.0 to about 5.0 (bit scores 54.6 to 148.4, e-values 1e^−05^ to 1e^−34^ on the NRDB). In this range, the difference in mean RIC was 0.13 higher for the model trained on electronic data than the model trained on experimental data (p <2.2e^−16^) on the training data set and 0.12 higher on the test set (p = 3.9e^−14^). The similar difference in mean RIC between the models for both training and test set provides some evidence that this result is not an artifact of a single data set. Another way to state this result is that the function similarity between proteins with comparable sequence similarity is *higher* on average when one has been electronically annotated than that observed between two experimentally characterized proteins. We did note a couple of cases in the electronic annotation data where no function similarity was indicated between two proteins but their sequence similarity was very high (log bit score >6.0). After inspecting some of these cases in more detail, we concluded that they were due to spurious electronic annotations (seeFile S1).

### Use Case 2: Annotation of Hypothetical Proteins

BLAST comparisons of hypothetical proteins versus the 738 experimentally characterized function proteins from our test and training data sets resulted in 58,038 BLAST alignments, after selecting only the best hit by bit score. These 58,038 hypothetical proteins were mapped to their Entrez Gene identifiers to account for splice variants and also for proteins marked as “removed” from the NCBI database (for unknown reasons). This resulted in 47,364 unique Entrez Genes with varying degrees of sequence similarity to experimentally characterized proteins (Table 2).

**Table 2:** Hypothetical proteins with at least some similarity to experimentally characterized proteins.

**RIC**	**0.10**	**0.25**	**0.50**	**0.75**	**0.90**	**0.95**
**# Proteins**	**47363**	**26613**	**16248**	**7544**	**2428**	**1349**

The columns represent the number of hypothetical proteins with an RIC greater than or equal to the stated RIC value. Although a large number of proteins have high function similarity to experimentally characterized proteins (RIC >0.90), the function similarity of the majority is rather moderate (RIC < = 0.50).

## Discussion

Statistical models are beneficial in data-rich environments such as 21^st^ century biology where they can be used to summarize and quantify biological relationships not readily apparent. The models used do not have to be overly complex, GLMs and GAMs are relatively simple to understand and deploy. What matters is that they are developed appropriately. In this study we use GLMs and GAMs to model the relationship between the sequence similarity between proteins and their function similarity. This represents a novel approach to functional annotation and potentially more accurate than current methods based on sequence similarity thresholds which do not account for the degree of function specificity which can be transferred between proteins over a wide range of sequence similarity. Our annotation model accounts for the fact that the function similarity between two proteins generally increases as their sequence similarity increases over a broad range of BLAST bit scores.

Using our annotation model we demonstrated that statistical models trained with experimental data generally predict lower functional similarity (measured by RIC), over a range of BLAST bit scores, than those trained with electronic data. This suggests that the sequence similarity threshold applied in many electronic annotations may be below the degree of sequence similarity required to transfer exact and specific functions from experimentally characterized proteins, at least for moderate bit score ranges (log bit score 4.0 to 5.0, see Figure 6). One implication of this is that many proteins with existing electronic annotations, at least those with specific functions, may be overly-specific. Overly-specific prediction, or simply over-prediction, is a common and “systematic” error of electronic annotations [1], although its exact prevalence is not known. We cannot extrapolate an error rate from our data for electronic annotations in public databases currently as we selected only specific electronic annotations (i.e. we did not analyze non-specific electronic annotations).

It's notoriously difficult to predict exact function in the “twilight zone” range of sequence similarity [29], [5], [6], [40] (i.e. moderate to low ranges of sequence similarity) but vitally important given the large volume of hypothetical proteins being discovered [29], [41], [17], [14]. Analyzing a fairly large sample of hypothetical proteins using our annotation model indicates that a general function can be predicted for a sizeable number. This represents a substantial improvement over their current state of annotation. Exact function predictions are problematic for these proteins however and our analysis indicates that direct transfer of functions will likely result in overly-specific function predictions due to their mostly moderate degree of sequence similarity to experimentally characterized proteins (Table 2). This illustrates the advantage, and novelty, of our annotation model. However, the degree of variability in the relationship between sequence similarity and function similarity in moderate sequence similarity ranges currently places constraints on the predictive accuracy of ours or any model. For instance, even in the bit score range of 3.5 to 4.0 a substantial proportion of proteins have an RIC of 0.0 as well as 1.0 (Figure 5). This may have something to do with the heterogeneity of functions in our data set and how the GO is constructed. A way to potentially address this is to create several annotation models each based on logically defined subsets of the GO (e.g. enzymes, receptors, etc.), similar to [39]. This would however require generation of much larger datasets.

An additional related problem is in regard to multi-domain proteins. Functions are reported at the protein level. Proteins however may be composed of multiple functional domains, or common and re-usable subsequences. Functions reported at the protein level may correspond to the whole protein, a subset of domains, or even a single domain. It can be hard to tell with certainty which single entity carries the function or if it's some sort of combination thereof. Inter-domain similarities regions of high similarity not functionally related due to incomplete (experimental) annotation, especially in the 3.5 to 5.0 bit score range, could be part of the reason for the high degree of variability in the relationship between bit score and RIC (Figure 5). In the future we intend to refine and develop computational methods to segment multi-domain proteins into their functional components in a reliable way. This may enable us to create much larger gold-standard data sets, with lower variability, with which to further develop, improve, and evaluate our model for protein functional annotation.

## Materials and Methods

### Gold-Standard Training and Test Data Sets

In order to avoid “circular logic”, or using predictions for prediction, training and test data sets only contained proteins with experimentally characterized functions. Many protein annotations are now attributed with GO evidence codes (http://www.geneontology.org/GO.evidence.shtml). These are a simple catalog of the type of evidence used when annotating a protein with a specific function. They can be used to filter out protein annotations not based on experimental evidence.

Proteins annotated with GO terms (molecular function ontology) were identified in the RefSeq and Uniprot databases [45]–[47]. Of these, only annotations attributed by GO evidence code “IDA”, indicating experimental evidence, were selected. The proteins from RefSeq and Uniprot were kept in two separate data sets which made up our training and test data sets respectively. This technique is known as split-sample model validation and is a robust method of model selection and validation [48]. To ensure independence between our data sets, proteins from the test set (Uniprot) determined to be identical or subsequences of proteins in the training set (RefSeq) were removed from the test set. Redundancy between proteins was determined using the *blastclust* (parameters: -p T –S 100) program made available with the downloadable BLAST program from the NCBI [44].

In addition to our training and test data sets we developed two additional model “use-case” data sets. The first was a compilation of RefSeq and Uniprot proteins electronically annotated (GO evidence code “IEA”) with identical GO terms as in our training and test set. A search of the Refseq database resulted in 1,870 proteins. A search for electronically annotated Uniprot proteins with identical GO terms as in our test set resulted in 90,480 proteins (electronic annotations are apparently more ubiquitous in Uniprot). The second use case data set was based on “hypothetical” proteins in RefSeq. A search of the Entrez database for RefSeq proteins on genomic sequence with “hypothetical” in the description line returned over two million protein sequences (June, 2009).

### Sequence Similarity

Local regions of similarity between all possible combinations of pair-wise protein sequence comparisons in the training and test data sets were determined by using BLAST [25]. Various statistics indicating the degree of similarity between two proteins were parsed out the BLAST results for each comparison including: 1) e-value, 2) bit score, 3) alignment length,4) number of alignment gaps, 5) amino acid identities, 6) amino acid positives, and 7) amino acid identities plus positives. We applied the log transformation to each of the BLAST statistics. The log transformation is often applied to biological data which vary across different orders of magnitude to make it more symmetrically distributed and thus more amenable to statistical modeling.

Sequence similarity data was creating using all-against-all BLAST comparisons within the training and test data sets, and between the training and test data sets and the use case data sets (redundant alignments were excluded). BLAST comparisons performed between the training and use case (electronic) data set yielded 13199 alignments. BLAST comparisons between the test and electronic Uniprot annotations resulted in over 400,000 alignments due to the much larger number of proteins (90,480). To make our analysis tractable, we took a sample of 5000 of these BLAST alignments. The sample contained a total 4781 Uniprot proteins with 77 GO terms, indicating an unbiased data set.

### Function Specificity and Functional Similarity

We use Information Content (IC) as a measure of GO term specificity. The IC of a GO term is related to the probability of discovering a particular GO term in a data set. The definition of IC is: 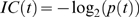
(1)


Where *t* is a particular GO term and *p* is the probability of that term occurring in a data set. The probability of a GO term is the number of times that it or any of its child terms occur in a data set. The approach of using IC as the term specificity measure in the GO is adapted from [43].

We use Relative Information Content (RIC) as a measure of the *function similarity* between GO terms (the term *semantic similarity* is also used [49]). RIC is used in the context of BLAST alignment between two proteins (with assigned GO terms), its formula is:
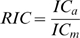
(2)


Where *IC_a_* is the IC of the ancestral GO term relating the GO terms assigned to two proteins in a BLAST alignment. The ancestral term found by traversing up the GO hierarchy. *IC*_m_ is the mean IC of the GO terms for the proteins in the BLAST alignment. A figure explaining IC and the GO can be found in Supporting Figure S1. Note that our RIC measure is an alternate derivation of Lin et al's semantic similarity measure between GO terms [50].

### Statistical Modeling and Software

We chose to model the relationship between sequence similarity and functional similarity using relatively straightforward Generalized Linear and Generalized Additive Models (GLM and GAM respectively). Statistical modeling was performed using the open-source R statistical package. The standard distribution includes packages for GLM's. A package for GAM's is provided by [48] and can be installed from the CRAN library (http://cran.r-project.org/). GLMs and GAMs are both statistical models which specify a relationship between predictor and response variables. GAMs are a generalization of GLMs in that predictor variables are not restricted to a linear relationship with some function of the response variable. A drawback of GAMs is their potential for over-fitting. For both types of models the response variable was restricted to 0.0–1.0 (all possible RIC values) using the logit transformation of the mean [51], and modeled it as a linear function of the predictor for the GLM model and as cubic splines for the GAM model. Parameter estimation can be achieved by using the quasi-likelihood function [48], which can be duplicated in R by using the “family = binomial” parameter for both GLM and GAM models. Predictor variables for both GLM and GAM models were selected using the “step” function in R which exhaustively adds or drops predictor variables from a statistical model according to the Akaike Information Criterion (AIC) [52]. Both GLM and GAM models were also fit using the single best BLAST predictor of RIC (bit score) as well as all reported BLAST similarity statistics. Quasi likelihood ratio tests were used for statistical comparison of developed models [51].

### Model Prediction Error

Model prediction error was determined by calculating Mean Squared Error (MSE) and Mean Residual Deviance (MRD). The calculation for MSE is:
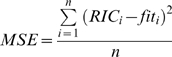
(3)


Where *RIC_i_* is the observed value (actual) and *fit_i_* is the value predicted for the same observed value from the model. The calculation for MRD is:
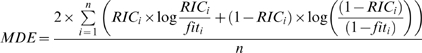
(4)


Where *RIC*_i_ is the observed RIC and *fit_i_* is the associated predicted RIC from the model. For RIC values of 0.0, a small number is added (1e^−07^) to avoid errors produced when taking the logarithm of 0.0. Both GLM and GAM models were fit to the training set using 10-fold cross-validation in order to estimate MSE and MRD in those cases. 10-fold cross-validation was used in order to remove the bias of estimating the error and training the model based on the same data.

## Supporting Information

Figure S1 An example of a hierarchical protein function description in the Gene Ontology (GO). The “protein phosphatase type 2A regulator activity” (PP2A) and “phosphatase inhibitor activity” (PIA) are relatively specific descriptions of protein function compared to the more general “protein phosphatase regulator activity” (PPRA) or completely non-specific root “molecular function” term. PPRA, and those terms further up in the hierarchy, are a common ancestral terms of PP2A and PIA. Both PP2A and PIA occur at a GO depth level 5 (counting from the root term) and are the same degree of specificity according to this metric. However, according to IC PP2A is a more specific function (IC = 13.7) compared to PIA (IC = 9.8) given the much lower number of proteins annotated with this function in the RefSeq database. Also note that IC decreases as the GO hierarchy is traversed upward. The PPRA GO term has IC = 8.8 for example, less than either PIA or PP2A. In general, the IC metric is a more normalized specificity metric than GO term depth. The RIC between PP2A and PIA, a measure their functional similarity, is calculated by obtaining the mean IC of PP2A and PIA (11.75), the IC of their most specific common ancestor term (PPRA, IC = 8.8), and taking the ratio of the ancestor and their mean IC (RIC = 8.8/11.75 = 0.75).
(0.68 MB TIF)

File S1 In depth-analysis of electronic annotation data.
(0.01 MB DOC)
