# Supplementary figures and images for: A Statistical Model of Protein Sequence Similarity and Function Similarity Reveals Overly-Specific Function Predictions
Source: PLoS One. 2009 Oct 21;4(10):e7546. doi: 10.1371/journal.pone.0007546 (PMC2760442; doi:10.1371/journal.pone.0007546)

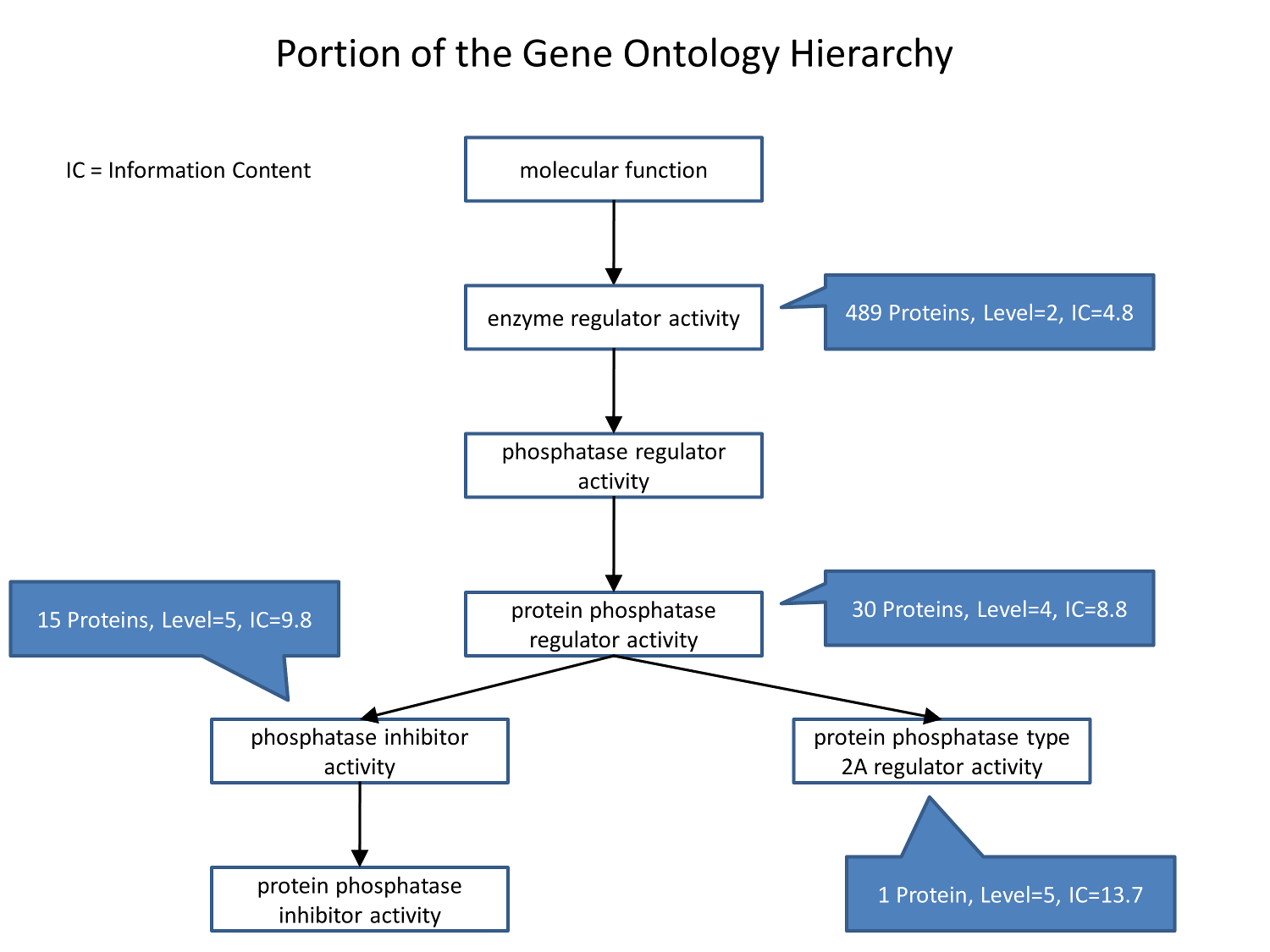

Supplement: Figure S1 — An example of a hierarchical protein function description in the Gene Ontology (GO). The “protein phosphatase type 2A regulator activity” (PP2A) and “phosphatase inhibitor activity” (PIA) are relatively specific descriptions of protein function compared to the more general “protein phosphatase regulator activity” (PPRA) or completely non-specific root “molecular function” term. PPRA, and those terms further up in the hierarchy, are a common ancestral terms of PP2A and PIA. Both PP2A and PIA occur at a GO depth level 5 (counting from the root term) and are the same degree of specificity according to this metric. However, according to IC PP2A is a more specific function (IC = 13.7) compared to PIA (IC = 9.8) given the much lower number of proteins annotated with this function in the RefSeq database. Also note that IC decreases as the GO hierarchy is traversed upward. The PPRA GO term has IC = 8.8 for example, less than either PIA or PP2A. In general, the IC metric is a more normalized specificity metric than GO term depth. The RIC between PP2A and PIA, a measure their functional similarity, is calculated by obtaining the mean IC of PP2A and PIA (11.75), the IC of their most specific common ancestor term (PPRA, IC = 8.8), and taking the ratio of the ancestor and their mean IC (RIC = 8.8/11.75 = 0.75). (0.68 MB TIF) [file pone.0007546.s001.tif]
