# Supplementary material for: A Statistical Model of Protein Sequence Similarity and Function Similarity Reveals Overly-Specific Function Predictions
Source: PLoS One. 2009 Oct 21;4(10):e7546. doi: 10.1371/journal.pone.0007546 (PMC2760442; doi:10.1371/journal.pone.0007546)
Supplement: File S1 — In depth-analysis of electronic annotation data. (0.01 MB DOC) [file pone.0007546.s002.rtf]

Supporting Information (File S1)

In depth-analysis of electronic annotation data.
In the electronic data set we identified a few cases where the similarity between two proteins was very high (bit score > 6.0) but exhibited no functional similarity (RIC = 0.0, Figure 4).  In our experimental data sets there were no cases where two proteins were not highly functionally related when the bit score between them was >6.0.  We investigated two of these cases further:
1.	Alignment:  NP_956956.1 and NP_067487.1
NP_956956.1 is the electronically annotated protein (GO:0003993, “acid phosphatase activity”) and hits very well to NP_067487.1 which is annotated with GO term (GO:0005487, “nucleocytoplasmic transporter activity”) by experimental evidence.  The gene name for both proteins is “nucleoporin 160”, indicating they may perform the same function although only one has experimental evidence.  NP_956956.1 also appears to be mis-annotated with the “acid phosphatase activity” function due to a spurious PROSITE pattern hit.  
2.	Alignment:  NP_990858.1 and NP_689597.1
Both proteins have the same gene symbol “Agrn” yet are annotated with different GO terms (GO:0043236 and GO:0030548 respectively).  However both are highly similar to another protein NP_786930.1 which is annotated with both terms (electronic evidence).  It is likely that both proteins should be annotated with both GO terms, although experimentation would be needed for validation.
